# COVID-19: risk factors for severe cases of the Delta variant

**DOI:** 10.18632/aging.203655

**Published:** 2021-10-28

**Authors:** Kaiyuan Hu, Liu Lin, Ying Liang, Xinning Shao, Zhongwei Hu, Hongbin Luo, Ming Lei

**Affiliations:** 1 Guangzhou Eighth People’s Hospital, Guangzhou Medical University, Guangzhou, China

**Keywords:** COVID-19, Delta variant, severe cases, clinical features, risk factors

## Abstract

Background: Since April 2021, the SARS-CoV-2 (B.1.167) Delta variant has been rampant worldwide. Recently, this variant has spread in Guangzhou, China. Our objective was to characterize the clinical features and risk factors of severe cases of the Delta variant in Guangzhou.

Methods: A total of 144 patients with the Delta variant were enrolled, and the data between the severe and non-severe groups were compared. Logistic regression methods and Cox multivariate regression analysis were used to investigate the risk factors of severe cases.

Results: The severity of the Delta variant was 11.1%. Each 1-year increase in age (OR, 1.089; 95% CI, 1.035–1.147; *P* = 0.001) and each 1-μmol/L increase in total bilirubin (OR, 1.198; 95% CI, 1.021–1.406; *P* = 0.039) were risk factors for severe cases. Moreover, the risk of progression to severe cases increased 13.444-fold and 3.922-fold when the age was greater than 58.5 years (HR, 13.444; 95% CI, 2.989–60.480; *P* = 0.001) or the total bilirubin level was greater than 7.23 μmol/L (HR, 3.922; 95% CI, 1.260–12.207; *P* = 0.018), respectively.

Conclusion: Older age and elevated total bilirubin were independent risk factors for severe cases of the Delta variant in Guangzhou, especially if the age was greater than 58.5 years or the total bilirubin level was greater than 7.23 μmol/L.

## INTRODUCTION

Up to June 24, 2021, more than 180 million cases have been diagnosed in more than 200 countries, with a mortality rate of about 2.17% [1]. Although many countries have started vaccinating, the virus mutates and new variants have been appearing, such as the Alpha variant in the UK and the Delta variant in India. Since the Delta variant was first identified in October 2020 in India, it has spread widely worldwide and is now the major epidemic strain [2, 3]. Due to strict control, no local COVID-19 cases have been found in Guangzhou, China, for more than one year. However, a new local case appeared in Guangzhou on May 21, 2021, and the number of diagnosis cases had exceeded 100 by June 14, 2021. Genetic analysis confirmed that the Delta variant strain caused this outbreak. This is the first indigenous COVID-19 outbreak in China caused by the Delta variant. The goal of this study was to investigate the clinical features and risk factors for severe cases with the Delta variant in Guangzhou. Monitoring these factors can help clinicians identify severe cases early and take subsequent interventions to control and reduce the illness.

## RESULTS

### Baseline characteristics

Among the 144 patients, 16 (11.1%) were in the severe group, and no patient died by the end of the study. The median age of all patients was 48.5 years (IQR 30.3–63.8), and 59.7% of the patients were female (Table 1). The most common comorbidity was hypertension (24, 16.7%), followed by diabetes (8, 5.6%) and cardiovascular disease (CVD; 5, 3.5%; Table 1). Twenty-six patients (18.1%) received one or two doses of the COVID-19 vaccine (Table 1). Cough (70, 48.6%) was the most common symptoms on admission, followed by fever (58, 40.3%), sputum (45, 31.3%), sore throat (24, 16.7%), headache (17, 11.8%), fatigue (16, 11.1%), and diarrhea (5, 3.5%; Table 1).

**Table 1:** Baseline characteristics of non-severe or severe patients of Delta variant in Guangzhou.

**Demographics and clinical characteristics**	**No. (%)**			***P* value**
	**Total (144)**	**Non-severe (128)**	**Severe (16)**	
Age, median (IQR), years	47.5 (30.3–63.8)	45 (25–59.5)	72 (63–83)	<0.001
Age groups (years):	..	..	..	<0.001
≤58.5	98 (68.1)	96 (75)	2 (12.5)	..
>58.5	46 (31.9)	32 (25)	14 (87.5)	
Sex:	..	..	..	1.000
Male	58 (40.3)	52 (40.6)	6 (37.5)	..
Female	86 (59.7)	76 (59.4)	10 (62.5)	..
Comorbidity:	..	..	..	..
Hypertension	24 (16.7)	17 (13.3)	7 (43.8)	0.006
Diabetes	8 (5.6)	6 (4.7)	2 (12.5)	0.479
CVD	5 (3.5)	4 (3.1)	1 (6.3)	1.000
Fever:	..	..	..	0.066
T < 37.3°C	86 (59.7)	77 (60.2)	9 (56.3)	..
37.3°C ≤ T < 38.1°C	26 (18.1)	24 (18.8)	2 (12.5)	..
38.1°C ≤ T < 39°C	26 (18.1)	24 (18.8)	2 (12.5)	..
39°C ≤ T	6 (4.2)	3 (2.3)	3 (18.8)	..
Cough	70 (48.6)	62 (48.4)	8 (50)	0.906
Sputum	45 (31.3)	39 (30.5)	6 (37.5)	0.567
Fatigue	16 (11.1)	13 (10.2)	3 (18.8)	0.542
Nausea and Vomiting	2 (1.4)	2 (1.6)	0 (0)	1.000
Diarrhea	5 (3.5)	4 (3.1)	1 (6.3)	1.000
Headache	17 (11.8)	16 (12.5)	1 (6.3)	0.749
Sore throat	24 (16.7)	19 (14.8)	5 (31.3)	0.192
Vaccination	26 (18.1)	26 (20.3)	0 (0)	0.100
Survival time	11 (6–14)	12 (8–14)	4 (4–5.8)	<0.001

Abbreviations: CVD: Cardiovascular disease; T: Temperature. *P* values indicate differences between Severe and Non-severe patients. *P* < 0.05 was considered statistically significant.

### Laboratory and radiological findings

Compared with non-severe patients, lymphocytes, platelets, and albumin were significantly reduced, whereas creatinine, C-reactive protein (CRP), total bilirubin, D-dimer, procalcitonin (PCT), and blood glucose were significantly increased in patients in the severe group (Table 2). Furthermore, the levels of nucleocapsid protein and open reading frame 1ab (ORF1ab) in the non-severe group were higher than in the severe group, indicating that the viral load was higher in the latter (Table 2). Thirty-one (21.5%) patients had unilateral pneumonia, and only two of them were severe cases; 64 (44.4%) patients had bilateral pneumonia, and 13 (81.3%) of these were severe cases (Table 2).

**Table 2:** Laboratory and radiological findings of non-severe or severe patients of Delta variant in Guangzhou.

	**Median (IQR)**			***P* value**	**Normal range**
	**Total (144)**	**Non-severe (128)**	**Severe (16)**		
Laboratory findings:	..	..	..	..	..
WBC (×109/L)	5.8 (4.6–6.9)	5. 8(4.7–7.0)	5.1 (4.2–6.3)	0.232	4–10
Lymphocyte count (×109/L)	1.1 (0.8–1.5)	1.1 (0.8–1.6)	1.0 (0.7–1.1)	0.013	1.1–3.2
Hemoglobin (g/L)	133 (125–145)	134 (126–145)	131 (123–147)	0.602	130–175
Platelet count (×109/L)	192 (156–241)	198 (160–245)	159 (135–195)	0.013	125–350
CRP (mg/L) (No (%)):	..	..	..	..	≤10
>10	46 (31.9)	34 (26.6)	12 (75)	<0.001	..
PCT (ng/mL)	0.06 (0.04–0.09)	0.06 (0.04–0.08)	0.10 (0.06–0.13)	0.015	<0.05
D-dimer (mg/L FEU)	0.31 (0.18–0.44)	0.28 (0.17–0.43)	0.44 (0.35–0.58)	0.001	<0.55
ALT (U/L)	14.3 (9.9–19.7)	14.0 (9.9–18.9)	15.1 (12.7–25.0)	0.416	9–50
AST(U/L)	20.1 (15.8–27.1)	19.6 (15.8–26.0)	20.8 (18.9–29.9)	0.352	15–40
Total bilirubin (μ mol/L)	6.5 (4.7–8.7)	6.5 (4.5–8.3)	8.2 (6.7–12.4)	0.009	3.4–17.1
Total bilirubin (μ mol/L) (No (%)):	..	..	..	0.002	..
>7.23	56 (38.9)	44 (34.4)	12 (75)	..	..
≤7.23	88 (61.1)	84 (65.6)	4 (25)	..	..
Albumin(g/L)	43.4 (39.9–46.3)	44.4 (40.6–46.6)	39.8 (36.5–42.0)	0.001	40–55
Creatinine (μ mol/L)	67.3 (55.0–78.9)	65.6 (54.0–76.1)	76.0 (68.1–93.9)	0.021	54–106
Creatinine kinase (U/L)	104 (76–149)	100 (77–148)	110 (72–232)	0.473	50–310
Uric Acid (μ mol/L)	307 (245–371)	308 (250–371)	291 (204–411)	0.629	180–420
Blood glucose (mmol/L)	6.1 (5.2–7.3)	6.0 (5.2–7.0)	8.5 (6.6–9.9)	0.001	3.9–6.1
Lactic acid (mmol/L)	1.4 (1.2–2.1)	1.4 (1.2–2.1)	1.3 (1.0–1.8)	0.324	0.5–1.7
Potassium (mmol/L)	3.5 (3.3–3.6)	3.5 (3.3–3.7)	3.3 (3.2–3.5)	0.065	3.5–5.5
RT-PCR:	..	..	..	..	..
Nucleocapsid protein	23.2 (19.5–28.4)	23.6 (19.8–28.4)	20.2 (18.5–29.1)	0.320	>40
ORF1ab	25.0 (20.7–30.0)	25.5 (21.0–30.0)	22.3 (19.5–29.6)	0.342	>40
Chest radiography findings:	..	..	..	0.005	..
Unilateral pneumonia	31 (21.5)	29 (22.7)	2 (12.5)	..	..
Bilateral pneumonia	64 (44.4)	51 (39.8)	13 (81.3)	..	..

Abbreviations: WBC: White blood cell; ALT: Alanine transaminase; AST: Aspartate aminotransferase; CRP: C-reactive protein; PCT: Procalcitonin; ORF1ab: open reading frame 1ab. *P* values indicate differences between Severe and Non-severe patients. *P* < 0.05 was considered statistically significant.

### Treatments and outcomes

Only 14 patients received antibiotics, including three non-severe patients and 11 severe patients. There was a significant difference between the two groups. Eleven patients received glucocorticoid, and 16 patients received gamma globulin. Of the non-severe patients, 41 patients did not receive oxygen, and 47 patients only received normal-flux oxygen, while all severe patients received high-flow oxygen. All severe patients received high flows through nasal cannulae therapy. With the exacerbation of the disease, seven severe patients received tracheal intubation. Furthermore, one of them was treated with a tracheotomy and extracorporeal membrane oxygenation (ECMO). As of June 14, 13 severe patients were admitted to the intensive care unit (ICU), compared with only three non-severe patients (Table 3).

**Table 3:** Treatments and outcomes of non-severe or severe patients of Delta variant in Guangzhou.

	**No. (%)**			***P* value**
	**Total (144)**	**Non-severe (128)**	**Severe (16)**	
Treatments:				
Antibiotics	14 (9.7)	3 (2.3)	11 (68.8)	<0.001
Glucocorticoid	11 (7.6)	4 (3.1)	7 (43.8)	<0.001
Gamma globulin	16 (11.1)	9 (7)	7 (43.8)	<0.001
Oxygen uptake:				
None	41 (28.5)	41 (32)	0 (0)	0.017
Normal-flux	47 (32.6)	47 (36.7)	0 (0)	0.003
High-flux	55 (38.9)	40 (31.3)	16 (100)	<0.001
High flows through nasal cannulae	52 (36.1)	36 (28.1)	16 (100)	<0.001
Tracheal intubation	7 (4.9)	0 (0)	7 (43.8)	<0.001
Tracheotomy	1 (0.7)	0 (0)	1 (6.3)	0.111
ECMO	1 (0.7)	0 (0)	1 (6.3)	0.111
Outcomes:				
ICU Admission	17 (11.8)	4 (3.1)	13 (81.3)	<0.001

Abbreviations: ECMO: Extracorporeal membrane oxygenation; ICU: intensive care unit. *P* values indicate differences between Severe and Non-severe patients. *P* < 0.05 was considered statistically significant.

### Univariate and multivariate analysis of risk factors of severe cases

Univariate logistic regression analysis found that age, hypertension, fever (39°C ≤ T), total bilirubin, CRP, lymphocytes, blood glucose, and bilateral pneumonia were related to severe cases (Table 4). All variables with significant statistical differences in the univariate logistic regression analysis were used to construct a multivariable logistic regression model. The results suggested that each 1-year increase in age (OR, 1.089; 95% CI, 1.035–1.147; *P* = 0.001), each 1-μmol/L increase in total bilirubin (OR, 1.198; 95% CI, 1.021–1.406; *P* = 0.039), and temperature equal to or greater than 39°C (OR, 25.292; 95% CI, 2.086–306.677; *P* = 0.011) were independent risk factors for severe cases (Table 4).

**Table 4:** Univariate and multivariate analysis of risk factors of severe cases in Guangzhou.

	**Univariable OR (95% CI)**	***P* value**	**Multivariable OR (95%) CI)**	***P* value**
Demographics and clinical characteristics				
Age, years	1.089 (1.043–1.137)	<0.001	1.089 (1.035–1.147)	0.001
Comorbidity present (vs not present)				
Hypertension	5.078 (1.671–15.438)	0.004	..	0.755
Fever:	..	0.071	..	0.091
T<37.3°C	1 (ref)	..	..	
37.3°C ≤T<38.1°C	0.713 (0.144–3.529)	0.678	..	0.783
38.1°C ≤T<39°C	0.713 (0.144–3.529)	0.678	..	0.721
39°C ≤T	8.556 (1.497–48.885)	0.016	25.292 (2.086–306.677)	0.011
Laboratory and radiography findings				
Lymphocyte count (×109/L)	0.223 (0.059–0.846)	0.027	..	0.076
Platelet count (×109/L)	0.987 (0.975–0.998)	0.019	..	0.256
CRP (mg/L) (No (%)):	..	..	..	..
>10	8.294 (2.504–27.472)	0.001	..	0.230
PCT (ng/mL)	2.748 (0.147–51.428)	0.499	..	..
D-dimer (mg/L FEU)	2.585 (0.789–8.471)	0.117	..	..
Total bilirubin (μ mol/L)	1.168 (1.029–1.326)	0.016	1.198 (1.021–1.406)	0.039
Albumin (g/L)	0.971 (0.934–1.009)	0.131	..	..
Creatinine (μ mol/L)	1.0 (0.996–1.003)	0.926	..	..
Blood glucose (mmol/L)	1.367 (1.125–1.662)	0.002	..	0.444
Potassium (mmol/L)	0.319 (0.076–1.342)	0.119	..	..
CT feature (vs no pneumonia)	..	0.023	..	0.514
Unilateral pneumonia	3.31 (0.287–38.144)	0.337	..	0.255
Bilateral pneumonia	12.235 (1.541–97.136)	0.018	..	0.317

Abbreviations: OR: odds ratio; T: Temperature. *P* values indicate differences between Severe and Non-severe patients. *P* < 0.05 was considered statistically significant.

### Kaplan-Meier and multivariate COX analysis of risk factors of severe cases

**Figure 1:**
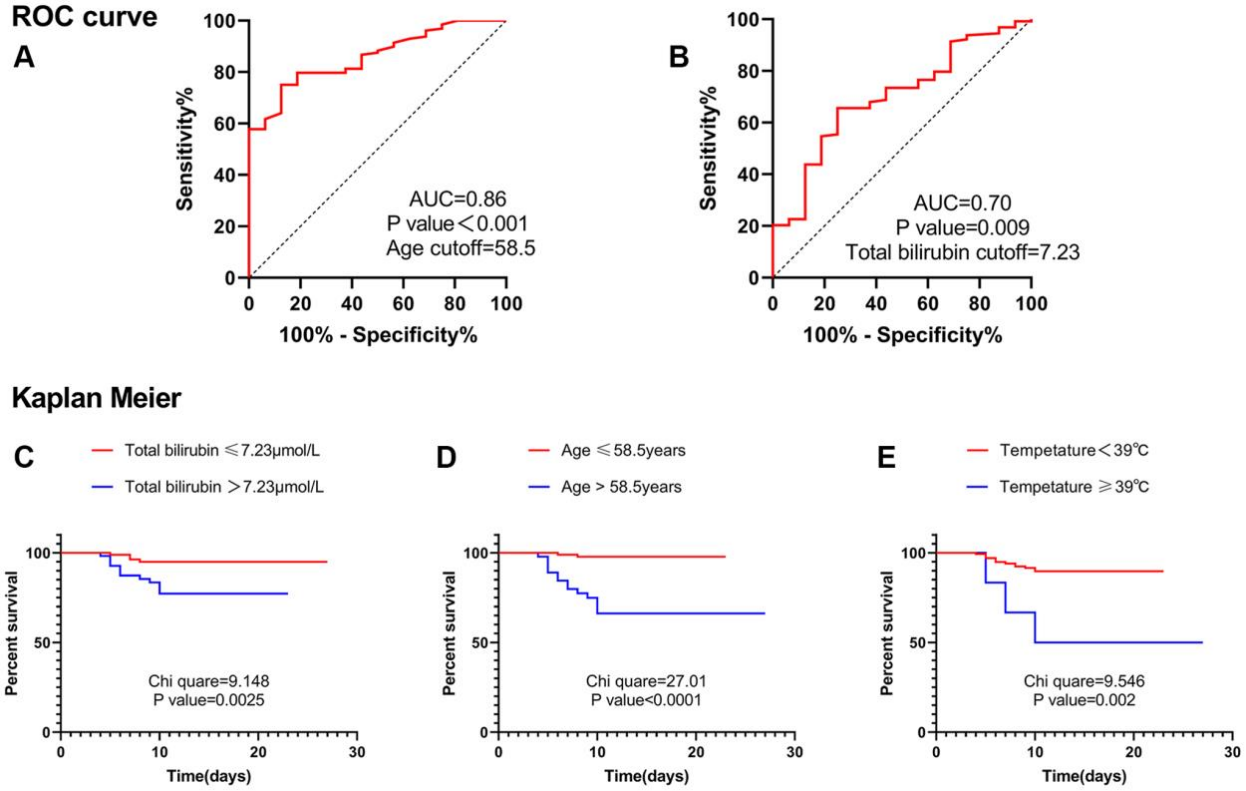
**ROC curve and Kaplan Meier.** (**A**) ROC curve suggested that age was valuable in predicting severe disease, and the cut-off point was 58.5 years. (**B**) ROC curve suggested that total bilirubin level was valuable in predicting severe disease, and the cut-off point was 7.23 μmol/L. (**C**) Kaplan Meier analysis suggested that patients with total bilirubin levels greater than 7.23 μmol/L were more likely to develop into severe cases. (**D**) Kaplan Meier analysis suggested that patients with age greater than 58.5 years were more likely to develop into severe cases. (**E**) Kaplan Meier analysis suggested that patients with Temperature ≥39°C were more likely to develop into severe cases. Abbreviation: AUC: area under the curve.

In the receiver operating characteristic (ROC) curve, the optimal cut-off value of age was 58.5 years, and the area under the curve (AUC) was 0.86 (Figure 1A). The optimal cut-off value of total bilirubin was 7.23 μmol/L, and the AUC was 0.70 (Figure 1B). Therefore, Kaplan– Meier analysis suggested that age greater than 58.5 years, total bilirubin greater than 7.23 μmol/L, and temperature equal to or greater than 39°C were risk factors for severe cases (Figure 1C–1E). Multivariate cox analysis was performed on the above three factors. The results suggested that age greater than 58.5 years (HR, 13.444; 95% CI, 2.989–60.480; *P* = 0.001) and total bilirubin greater than 7.23 μmol/L (HR, 3.922; 95% CI, 1.260–12.207; *P* = 0.018) were independent risk factors for severe cases (Table 5).

**Table 5:** Multivariate COX analysis of risk factors of severe cases in Guangzhou.

	**HR (95% CI)**	***P* value**
Age (>58.5years)	13.444 (2.989–60.480)	0.001
Fever (≥39°C)	3.126 (0.876–11.155)	0.079
Total bilirubin (>7.23 μ mol/L)	3.922 (1.260–12.207)	0.018

Abbreviation: HR: hazard ratio.

## DISCUSSION

In late 2019, SARS-CoV-2 broke out in Wuhan, with a severity rate as high as 31.7% in the early days [4] and eventually caused thousands of deaths in the city. The outbreak quickly spread to Guangzhou, where about 300 people were infected [5]. At that time, the severity rate in Guangzhou was 10.4%, and the mortality rate was 0.3% [5]. SARS-CoV-2 has mutated many times subsequently. Recently, the SARS-CoV-2 (B.1.167) Delta variant has raged around the world. In India, due to the widespread presence of the Delta variant, the number of COVID-19 deaths has risen from 100 to 200 deaths per day in the earlier months to about 4,000 deaths per day [1]. In the UK, in the week of June 2, about 34% of people with the Delta variant visited the emergency departments of hospitals and were admitted at hospitals overnight [2]. Influenced by SARS-CoV-2 (B.1.167), more than 100 indigenous cases were reported in Guangzhou again. It began with an elderly lady and quickly spread to the community through her family. Compared with the epidemic caused by SARS-CoV-2 in Guangzhou last year, the current epidemic caused by SARS-CoV-2 (B.1.167) has the following characteristics: (1) short incubation period, 2–4 days; (2) fast propagation speed; (3) strong ability of infection; and (4) high viral load. These characteristics were the same as those reported in other countries [1, 2]. Up to June 14, of the 144 patients in our database, none died, and the severity of the Delta variant in Guangzhou was 11.4%.

It was interesting to find that patients with elevated total bilirubin had a higher risk of becoming severe cases. In our study, when the total bilirubin level was greater than 7.23 μmol/L, the risk of severe illness increased 3.922-fold. Although three patients’ had total bilirubin levels higher than normal, clinicians tend to ignore this indicator. Studies have shown that some COVID-19 patients have elevated total bilirubin [6, 7]. Liang W et al. reported that direct bilirubin level was an independent risk factor for patients with severe COVID-19 symptoms [8]. One study showed that elevated bilirubin levels were an independent risk factor for severe cases of COVID-19 [9]. Another study of COVID-19 patients in Shenzhen—and this was caused by SARS-CoV-2—showed that total bilirubin levels were higher in severe cases [10], but that relationship was not identified in the Guangzhou study [5]. We have found this relationship in Guangzhou patients with COVID-19 caused by the Delta variant strain. Therefore, clinicians need to pay more attention to the patient’s total bilirubin levels, even if the levels are not above normal.

Total bilirubin elevation was mainly seen in several cases of liver cell damage, cholestasis, and hemolysis. SARS-CoV-2 could use transmembrane serine protease 2 (TMPRSS2) as a docking and entry receptor on host cells [11]. *TMPRSS2* mRNA expression was found in hepatocytes [12]. Electron microscope analysis of liver samples from two deceased COVID-19 patients with elevated liver enzymes revealed the presence of intact virus particles in the cytoplasm of hepatocytes [13]. The SARS-CoV-2 infection has been observed in small intestinal organoids [14, 15]. The virus replicates rapidly in the gut and could reach the liver through portal vein circulation. Hepatic Kupffer cells might trigger an inflammatory response as they attempt to clear the virus [7]. Early over-release of inflammatory cytokines in COVID-19 patients was associated with disease severity and might cause cytokine storm syndrome (CSS) [16]. CSS might cause disseminated intravascular coagulation (DIC) [17] and was associated with multiple organ failure (MOF). Microthrombotic endodermatitis and MOF-associated hepatic ischemia might cause hepatocyte injury. In addition, acetaminophen, antibiotics, corticosteroids, and immunomodulators used in treating COVID-19 patients had potential hepatotoxicity. Hypoxic hepatitis caused by hemodynamics and oxygen delivery changes could also lead to liver cell damage [7]. In postmortem assessments of COVID-19 patients, cholestasis features such as bile duct hyperplasia and inflammatory portal infiltration have been reported [18, 19]. Studies suggested that the following factors might hit bile ducts in COVID-19 patients: hypoxia due to respiratory failure or systemic inflammatory response syndrome (SIRS) leads to the spread of inflammation and fibrosis and potentially viral infection of bile duct cells [20]. Autoimmune hemolytic anemia (AIHA) with SARS-CoV-2 infection may cause hemolysis [21], but no AIHA patients were included in this study. The severe patients we followed had a higher viral load than non-severe patients. In addition, severe patients had a higher rate of antibiotics use, corticosteroids, and more obvious hypoxia, which were potential factors for liver injury or cholestasis, although these injuries were not apparent. This could well explain why the total bilirubin levels are higher in severe patients than in non-severe patients.

A wealth of evidence suggested that age itself was the most important risk factor for severe COVID-19 disease [22–24]. A study showed that age (OR, 1.057; 95% CI, 1.018–1.098; *P* = 0.004) was an independent risk factor for severe disease in patients with COVID-19 in Guangzhou last year [5]. Another study showed that older COVID-19 patients have more atypical symptoms, with increased comorbidities, organ injuries, secondary infection, immunodeficiency, and a higher risk of critical illness [25]. Studies from China, the United States, and Italy have shown that COVID-19 patients of different ages have different severe illness and mortality rates. Generally speaking, the older the patients were, the higher the mortality rate was [26–28]. Some previous studies might explain this. First, immune-senescence and inflammation play a major role in making older patients more vulnerable to severe COVID-19 outcomes [29, 30]. Second, Mikhail V. Blagosklonny [31] proposed that the hyperfunction theory of quasi-programmed aging explains the correlation between the age of the COVID-19 patient and the mortality rate. Because aging was driven by inappropriately high cellular functioning, cellular hyperfunctions may eventually switch to cellular exhaustion and loss of functions at late stages. Third, biological age was more relevant to COVID-19 than the chronological age, and epigenetic clocks and glycosylation clocks were biomarkers for objective estimates of biological age [30, 32]. Age-related epigenome changes have important implications for the immune system, including macrophage pattern recognition, cytokine production, and T cell function. Moreover, changes in glycosylation during aging might make older people more susceptible to severe COVID-19 [30]. Fourth, the dysregulation of the epigenome was closely associated with chronic disease states and aging. The composition and function of immune cells can be impaired by age-related host epigenome changes [30]. Finally, the known mislocation of SIRT1 and SIRT6 throughout the genome and the decline of NAD+ during aging could be major factors causing age-dependent symptoms of COVID-19 [30]. Age was also one of the risk factors for severe patients in our study cohort. In particular, the risk of severe cases increased 13.444-fold when the patient was older than 58.5 years. Therefore, older patients need to have an early diagnosis and their systemic comorbidities treated carefully.

Fever was the most common symptom in these patients, followed by cough, sore throat, fatigue, expectoration, headache, and other symptoms. This trend was similar to that of previous studies [5, 33, 34]. It was suggested that some symptoms might be the independent risk factors for patients to develop into severe cases [9]. In our study, multivariate logistic regression analysis suggested that a temperature equal to or greater than 39°C was a risk factor for developing severe disease. However, in multivariate Cox regression analysis, temperature ≥39°C was excluded. According to clinical observations, some patients had no fever symptoms until 1–2 days after hospitalization; yet, the data used in these statistical analyses were registered at admission. Overall, a temperature ≥39°C remains a key concern for clinicians.

Except for age and total bilirubin, studies have shown that comorbidity, such as diabetes, and CVD, laboratory indicators, such as lymphocyte count, white blood cell count, platelet count, blood glucose, PCT, and D-dimer, and imaging findings, such as double pneumonia or severe pulmonary inflammation, were independent risk factors for severe disease and poor prognosis [35–42]. However, in our study, these relationships were not found. This might be related to the lack of a sufficient number of samples or the variant of the virus. Further studies are needed to clarify this issue.

There is currently no specific drug for COVID-19 patients. Some early studies reported that chloroquine, hydroxychloroquine, umifenovir (arbidol), redeliver, and traditional Chinese medicine might be effective [43–48], but recent research suggested the opposite [49–51]. Vaccines were the most effective means of prevention [52], although the Delta variant is moderately resistant to vaccines, especially in people who have received only one dose [53]. Twenty-six patients in our study were infected after receiving the vaccine, but none developed severe disease.

The study has several limitations. First, the lack of laboratory data on serum cytokines, chemokines, and other factors makes it impossible to assess the levels of inflammation and cytokine storms in these patients. Second, this study lacked bile acid, hepatobiliary ultrasound, and even liver biopsy data; so, it could not further clarify the relationship between total bilirubin elevation and severe patients. Third, this is a retrospective study, and the data collected are only a preliminary assessment of the clinical characteristics and risk factors of severe cases of COVID-19. Further research is still needed.

In conclusion, our study showed that the mortality and severity of the Delta variant in Guangzhou were much lower than in the rest of the world. The risk factors for severe cases of the Delta variant in Guangzhou included older age and elevated total bilirubin, especially if the age was greater than 58.5 years or the total bilirubin level was greater than 7.23 μmol/L. Investigating and monitoring these factors can help clinicians identify patients with poor prognoses early and initiate aggressive interventions that benefit patients and reduce the severity and mortality. Our research also provided significant experience and reference for countries around the world to fight against the Delta variant.

## MATERIALS AND METHODS

### Study design and participants

This study was conducted retrospectively at Guangzhou Eighth People’s Hospital, the designated COVID-19 hospital in Guangzhou, China. From May 21, 2021, to June 11, 2021, 144 patients with the Delta variant were recruited. The end of the follow-up was June 14, 2021.

The study was approved by the Ethics Committee, and informed consent was obtained from all patients enrolled.

### Definitions

Based on the Chinese diagnosis and treatment guideline for COVID-19 (trial version 8.0) [54], 144 patients were divided into two groups: the non-severe group (128 cases), including light and general patients, and the severe group (16 cases), including severe and critical patients. A case was defined as severe if it met any of the following: (1) shortness of breath, respiratory rate ≥30 times/minute; (2) blood oxygen saturation ≤93% at rest; (3) oxygenation index (PaO2/FiO2) ≤300 mmHg; (4) pulmonary infiltrates >50% of the lung lesions within 24–48 hours; (5) respiratory failure, requiring mechanical ventilation; (6) shock; (7) multiple organ dysfunction, needing ICU monitoring treatment. In this study, the reference ranges of all laboratory test indicators were based on the laboratory of Guangzhou Eighth People’s Hospital.

### Data collection

The nursing records, clinical electronic medical records, laboratory tests, and radiological findings of the 144 patients, who were confirmed by nucleic acid testing and genetic analysis to have been infected by the Delta variant, were reviewed. In addition, the epidemiology, demographics, clinical manifestations, laboratory data, and computerized chest tomography (CT) findings were extracted for statistical analysis and research.

### Statistical analysis

Continuous variables were presented as median (interquartile range), and categorical variables were presented as numbers (%). We used a two-sample *t*-test or the Mann–Whitney *U* test to assess the differences between severe and non-severe groups for continuous variables and used the χ^2^ test or Fisher’s exact test to assess categorical variables. Univariate logistics regression analysis with a *P*-value <0.1 is presented in Tables 1 and 2. Then, multivariate logistic regression analysis was performed on the indicators with *P* < 0.05 in univariate logistic regression analysis. The cut-off was obtained through the ROC curve. Survival time was counted from the onset of the disease until the patient’s condition progressed to severe or until June 14. Kaplan–Meier and Cox regression analysis further investigated the risk factors of severe patients. Statistical analyses were performed using SPSS, ver. 22.0. *P* < 0.05 was considered statistically significant.
